# Administration of pentoxifylline to improve anemia of hemodialysis patients

**DOI:** 10.15171/jrip.2017.11

**Published:** 2016-11-26

**Authors:** Heshmatolah Shahbazian, Ali Ghorbani, Azita Zafar-Mohtashami, Abdolreza Balali, Armaghan AleAli, Gholam Reza Lashkarara

**Affiliations:** ^1^Diabetes Research Center, Ahvaz Jundishapur University of Medical Sciences, Ahvaz, Iran; ^2^Department of Internal Medicine, Shahid Rahimi Hospital, Lorestan University of Medical Sciences, Khoramabad, Iran; ^3^Ahvaz Jundishapur University of Medical Sciences, Ahvaz, Iran; ^4^Lorestan University of Medical Sciences, Khoramabad, Iran

**Keywords:** Anemia, Pentoxifylline, End-stage renal disease, Cytokine

## Abstract

**Introduction:** One of the most important causes of erythropoietin-resistant anemia in end-stage renal disease (ESRD) patients is increased levels of inflammatory cytokines.

**Objectives:** In this study pentoxifylline, an anti-inflammatory and anti-cytokine drug, with no significant side effects was used to manage anemia in ESRD patients.

**Patients and Methods:** Thirty-nine ESRD patients with erythropoietin-resistant anemia were assigned to two groups, the treatment and the control groups. In treatment group, 19 patients received erythropoietin, venofer and pentoxifylline for 6 months.

Patients in control group received erythropoietin and venofer. Hemoglobin (Hb), hematocrit (Hct), albumin and quantitative C-reactive protein (CRP) were measured at the beginning of the study, monthly and at the end of the study.

**Results:** Hb and Hct were significantly increased in the treatment group (9.33±1.25 g/dL and 28.08±3.88% at baseline; 11.22 ± 1.26 g/dL and 34.02 ± 3.72% at sixth month, *P* = 0.01), but not in the control group. CRP was significantly decreased in the treatment group but no significant change occurred in the control group.

**Conclusion:** Pentoxifylline is effective in improvement of erythropoietin-resistant anemia in ESRD patients.

**Implication for health policy/practice/research/medical education:**

Pentoxifylline is effective in improvement of erythropoietin-resistant anemia in ESRD patients.

## Introduction

Chronic kidney disease (CKD) is defined as persistent disorder in kidney activities in maintaining normal levels of protein metabolism products such as urea, blood pressure, hematocrit (Hct), and acid-base and fluid and electrolytes equilibrium (1). Clinically, the disease is diagnosed with progressive disorder in kidney function that will eventually lead to renal failure. The end-stage of renal failure which needs renal replacement such as dialysis or transplantation is called end-stage renal disease (ESRD). According to the World Health Organization (WHO) with the increasing incidence and prevalence of diabetes and the relationship between diabetes and kidney disease, in the near future we will encounter a significant increase in kidney disease (2). According to the latest data, there are about 15000 ESRD patients in Iran, around 53 cases in one million people. Around half of them are treated with hemodialysis. Many of newly diagnosed CKD patients have at least temporarily undergone hemodialysis (3). Anemia is directly related to azotemia, with reaching creatinine to 3 mg/dL, anemia can be detected in patient laboratory exams (4). According to WHO criteria, 90% of CKD patients with glomerular filtration rate (GFR) <25-30 mg/dL have anemia and in many of them hemoglobin (Hb) level is less than 10 g/dL (5). The main cause of anemia in CKD is insufficient production of erythropoietin by kidney. Other causes are iron deficiency, folate deficiency, severe hyperparathyroidism with bone marrow fibrosis and low serum vitamin B12 (1).

About 65% of patients have Hb equal or less than 11 g/dL. The risk of mortality will be less, if anemia is treated before commencement of dialysis (6,7). Anemia has many symptoms and signs such as fatigue, depression, dyspnea, palpitation, left ventricular hypertrophy and left ventricular systolic dysfunction (8). Anemia will increase the risk of morbidity and mortality particularly due to cardiovascular or cerebrovascular diseases (6-9).

Alpha epoetin (EPO) and some other similar drugs have become the standard treatment for anemia in CKD patients (5). But resistance to these drugs is increasing due to chronic inflammation in these patients.

Pentoxifylline is commonly used for peripheral vascular diseases but it may also have anti-inflammatory effects (10). It prevents the production of tumor necrosis factor-alpha (TNF-α) in lymphocytes and reducing TNF-α and C-reactive protein (CRP) in circulatory system. These effects have been useful in managing idiopathic cardiomyopathy, nephrotic syndrome, systemic vasculitis and rheumatoid arthritis (11).

The most side effects of the drug are gastrointestinal ones such as nausea and vomiting in 1%-2% of patients. Anaphylactoid reactions, angioedema, angina pectoris, and other side effects have been rarely reported (12).

## Objectives

Pentoxifylline may have some effects in increasing Hb level in CKD patients (13). Thus, it is our objective to determine this effect on hemodialysis patients in whom anemia is very common despite of treatment with erythropoietin.

## Patients and Methods

This is a clinical trial study involving ESRD patients of Golestan and Imam Khomeini hospitals of Ahvaz who were at least one year under hemodialysis. The inclusion criteria were Hb <11 g/dL, taking 9000-12000 IU/week erythropoietin, Kt/V>1.2, administration of adequate doses of venofer. Exclusion criteria were iron deficiency with serum ferritin <100 µg/dL and transferrin saturation <20%, intact parathyroid hormone (iPTH) > 300 ρg/mL, total hours of dialysis less than 12 in a week, history of hematemesis, melena or occult blood in stool, history of angina pectoris, history of recent cerebral or retinal hemorrhage, simultaneous infectious or rheumatologic diseases, sensitivity to theophylline or other xanthine. All the necessary information about the study was explained to patients and written consent was taken from each patient.

According to the results of other studies (14) and with 90% power and α = 0.05, 19 patients were calculated to be in each group. During recruitment from September 2009 to November 2009, 51 out of 100 patients found to have the inclusion criteria. Forty-three of them were randomly chosen and randomly assigned to two groups, 21 in treatment group (taking pentoxifylline 400 mg daily) and 22 in control group. Duration of the study was 6 months; patients were weekly controlled for side effects of the drug and tolerance of the drug; Kt/V of dialysis was checked monthly to be adequate. Patients with severe side effects of nausea and emesis were excluded from the study. Quantitative CRP levels were measured at the beginning and at the end of the study and Hb, Hct, albumin and Kt/V were measured monthly.

At the baseline, Hb, Hct, albumin and CRP were measured before starting dialysis and a blood sample was taken after dialysis for all patients for calculating Kt/V. Then oral pentoxifylline, 400 mg/d (as Apo-pentoxiphylline tablet 400 mg, slow release manufactured by APOTEX, Canada) was begun for the treatment group. For both group, Hb, Hct, albumin and quantitative CRP were measured monthly and at the end of the study.

### Ethical issues

The research followed the tenets of the Declaration of Hel­sinki; informed consent was obtained; and the research was approved by the ethical committee of Ahvaz Jundishapur University of Medical Sciences (Ethical code: 8467).

### Statistical analysis

The quantitative variables were presented as mean± standard deviation (SD) and compared by *t* test. The categorical variables were statistically analyzed by chi-square test. SPSS software was used for analysis and *P* values less than 0.05 were considered significant.

## Results

Baseline data are shown in Table 1. Two patients in the treatment group were excluded from the study due to drug side effects. Also in the control group, two patients were excluded from the study due to emigration. Therefore, treatment group consisted of 19 patients and control group of 20 patients (Table 2).

**Table 1:** Baseline data of treatment and control groups

**Variable**	**Treatment group**	**Control group**	***P*** ** value**
Age (years)	51.16 ± 12.84	52.85 ± 12.33	0.67
Sex			0.91
Male	11	11	
Female	10	11	
Duration of ESRD (years)	2.92 ± 1.91	4.08 ± 2.02	0.076
Serum ferritin (µg/dL)	432.37 ± 108.13	331.50 ± 167.6	0.98
iPTH (ρg/dL)	187.37 ± 96.93	164.5 ± 84.64	0.58
Hb (g/dL)	9.33 ± 1.25	9.73 ± 1.10	0.28
Hct (%)	28.08 ± 3.88	29.45 ± 3.76	0.27
Albumin (g/dL)	4.12 ± 0.37	3.79 ± 0.51	0.69
Quantitative CRP (mg/dL)	16.2 ± 12.86	9.54 ± 7.08	0.051
Kt/V	1.76 ± 0.18	1.53 ± 0.20	0.17

Abbreviations: ESRD, end-stage renal disease; CRP, C-reactive protein; Hb, hemoglobin; Hct, hematocrit; iPTH, intact parathyroid hormone.

**Table 2:** Comparing baseline and end results for treatment group and control group separately

**Variable**	**Treatment group**			**Control group**		
	**Baseline**	**End of study**	***P*** ** value**	**Baseline**	**End of study**	***P*** ** value**
Hb (g/dL)	16.2	11.22 ± 1.26	0.01	9.73 ± 1.10	9.77 ± 1.08	0.92
Hct (%)	28.08 ± 3.8	34.02 ± 3.72	0.01	29.45 ± 3.76	30.54 ± 3.83	0.39
CRP (mg/dL)	16.2 ± 12.89	7.53 ± 5.18	0.001	9.54 ± 7.08	9.66 ± 6.8	0.81
Albumin (g/dL)	4.12 ± 0.37	4.76 ± 0.4	0.02	3.76 ± 0.51	3.66 ± 0.49	0.54

Abbreviations: CRP, C-reactive protein; Hb, hemoglobin; Hct, hematocrit.

In treatment group, no difference of Hb and Hct between males and females after 6 months of treatment with pentoxifylline was detected (*P*>0.05).

The changes in the values of the variables in treatment group during 6 months were all significantly different from that of control group (Table 3).

**Table 3:** Comparing the end results of the treatment group and control group

**Variable**	**At the end of the study**		***P*** ** value**
	**Treatment group**	**Control group**	
Hb (g/dL)	11.22 ± 1.26	9.77 ± 1.08	0.001
Hct (%)	34.02 ± 3.72	30.54 ± 3.83	0.007
CRP (mg/dL)	7.53 ± 5.18	9.66 ± 6.8	0.28
Albumin (g/dL)	4.76 ± 0.4	3.66 ± 0.49	0.001

## Discussion

Anemia is one of the most common problems in ESRD. Recently, inflammatory processes have been proposed as the main causes of erythropoietin resistant anemia in ESRD (15,16). Inflammation is recognized as increase in concentration of positive acute phase proteins such as CRP (reaching as high as 5-10 mg/dL in renal failure) and decrease in negative acute phase proteins such as albumin and transferrin (17,18). This study revealed that 96% of ESRD patients were anemic and 53% of them, in spite of taking sufficient doses of erythropoietin, were still became anemic. The increase in Hb and Hct in treatment group was significant (*P*=0.01 and *P*=0.01 respectively), but not in control group. The decrease in mean quantitative serum CRP in treatment group was significant (*P*=0.001). Maybe the inflammation control has been important in improvement of anemia.

Albumin increased significantly (*P*=0.02) in treatment group. Albumin is a reactive in negative acute phase. Its increment indicates control of inflammation and improvement of anemia in these patients.

In 1999, Navarro and colleagues studied 7 ESRD patients treated with 400 mg oral pentoxifylline for 6 months and compared them with the control group. At the end of the study they showed significant increase in Hb and Hct amounts in the treatment group. They also showed significant decrease in TNF-α in the treatment group (*P*=0.01) (14).

Cooper and colleagues studied 16 ESRD patients (12 patients completed the study) with administration of 400 mg oral pentoxifylline for 4 months and showed significant increase in Hb (*P*=0.0001) and significant decrease in TNF-α (*P*=0.0007) and IFN-γ (*P*=0.002). The limitations of their study were no comparison with a control group, short duration of the study and low proportion of patients (19).

Johnson et al studied 62 hemodialysis patients, in two groups of 31 patients, a treatment group taking 400 mg oral pentoxifylline for 3 months and a control group. At the end of the study, Hb increased and inflammatory cytokines and CRP decreased significantly (20).

This study revealed that 400 mg/d of pentoxifylline controlled inflammatory cytokines and improvement of anemia. It was a double-blind study with appropriate proportion of patients however, its duration was short.

All the above mentioned studies revealed significant increase in Hb and Hct in ESRD patients after taking oral pentoxifylline.

In our study, significant increase in Hb and Hct in ESRD patients was demonstrated in the treatment group after taking pentoxifylline.

## Conclusion

According to the results of this study, it can be concluded that the administration of pentoxifylline 400 mg daily for 6 months to hemodialysis patients can control inflammation and thus improve anemia. It has no significant side effects and no effects on mortality of the patients.

## Limitations of the study

We suggest multi-centric studies on the impact of pentoxifylline on anemia of hemodialysis patents. One of the limitations of our study was a short duration of investigation. Thus larger studies with longer duration are suggested.

## Acknowledgements

The authors thank the deputy for research and Diabetes Research Center of Ahvaz Jundishapour University of Medical Sciences for both funding and supporting this research study.

## Authors’ contribution

HS and AZ participated in designing the protocol, statistical analysis and preparing the manuscript. AG and AB coordinated the collection of data. AZ and AG designed the research plan and organized the study. GL and AA performed analysis and interpretation of data. AZ prepared the final manuscript of the article.

## Conflicts of interest

The authors declare no conflict of interest.

## Ethical considerations

Ethical issues (including plagiarism, data fabrication, double publication) have been completely observed by the authors.

## Funding/Support

This article is extracted from Abdolreza Balali’s internal medicine dissertation. The study was supported by a grant from Ahvaz Jundishapur University of Medical Sciences (#U-87078, 2009).
